# Naphthenic Acids: Formation, Role in Emulsion Stability, and Recent Advances in Mass Spectrometry-Based Analytical Methods

**DOI:** 10.1155/2021/6078084

**Published:** 2021-12-17

**Authors:** Roselaine Facanali, Nathália de A. Porto, Juliana Crucello, Rogerio M. Carvalho, Boniek G. Vaz, Leandro W. Hantao

**Affiliations:** ^1^Institute of Chemistry, University of Campinas, Campinas 13083-862, SP, Brazil; ^2^Leopoldo Américo Miguez de Mello Research and Development Center, Petrobras, Rio de Janeiro 20031-912, RJ, Brazil; ^3^Institute of Chemistry, Federal University of Goiás, Goiânia 74690-900, GO, Brazil

## Abstract

Naphthenic acids (NAs) are compounds naturally present in most petroleum sources comprised of complex mixtures with a highly variable composition depending on their origin. Their occurrence in crude oil can cause severe corrosion problems and catalysts deactivation, decreasing oil quality and consequently impacting its productivity and economic value. NAs structures also allow them to behave as surfactants, causing the formation and stabilization of emulsions. In face of the ongoing challenge of treatment of water-in-oil (W/O) or oil-in-water (O/W) emulsions in the oil and gas industry, it is important to understand how NAs act in emulsified systems and which acids are present in the interface. Considering that, this review describes the properties of NAs, their role in the formation and stability of oil emulsions, and the modern analytical methods used for the qualitative analysis of such acids.

## 1. Introduction

In petroleum fields, crude oil and produced water (PW) emulsions are formed during oil production when the fluid undergoes flow restrictions. The inevitable emulsification of crude oil and PW may increase fluid viscosity, leading to several problems in flow assurance and primary processing. Emulsion stability is mainly determined by the structural properties of its interfacial layer [1–3].

Being among the most important surfactants naturally found in oil sources [4], naphthenic acids (NAs) comprise all petroleum-derived organic acids [5], including aromatics, tricyclic diamondoids, and sulfur-containing compounds [6]. For these compounds can migrate to water during the oil and bitumen extraction process [7], NAs are also detected in PW samples. The classes and concentration levels of NAs will vary depending on the origin of the oil [3], just as NAs and their salts may exhibit varying solubility in aqueous phases depending on their chemical structures [8, 9]. According to pH conditions and water content, acidic oils can form different types of emulsions with varying stability. However, emulsions are impacted by a multivariate and complex interplay of parameters and mechanisms, thus hindering the full comprehension of the role of NAs in stabilizing emulsions.

## 2. Emulsion

When flowing through pipes, oil and water are subjected to agitation, promoting dispersion from one phase to another and resulting in emulsions [10]. Defined as a mixture of two immiscible or partially immiscible liquids, where one liquid is dispersed in the other in the form of droplets of microscopic or colloidal size [10–12], emulsions can be found in almost all stages of the production chain, from exploration to refining [10].

**Figure 1:**
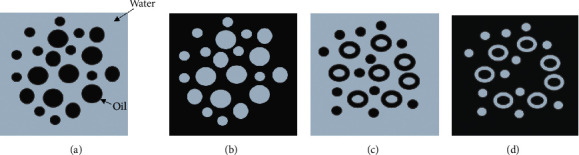
Representation of emulsions in which water is the continuous phase (a), oil is the continuous phase (b), and multiple systems (c, d). (a) Oil-in-water (O/W). (b) Water-in-oil (W/O). (c) Water-in-oil-in-water (W/O/W). (d) Oil-in-water-in-oil (O/W/O).

Emulsions are complex structures characterized according to the liquid that forms the continuous phase [13]: if the continuous medium is water, the emulsion is characterized as an oil-in-water type (O/W); conversely, if the continuous medium is oil, emulsion is named water-in-oil (W/O). In more complex situations, multiple water-in-oil-in-water (W/O/W) or oil-in-water-in-oil (O/W/O) systems are possible [10], as shown in Figure 1.

In the petroleum industry, W/O is the most common type of emulsion formed during oil production. Such emulsions typically contain ≤50% of water [14]. One may also find O/W emulsions, whose water content may be up to ≥80% [14].

Kinetically stable emulsions are formed in the presence of surfactants, whose hydrophilic-lipophilic balance (HLB) is the most important parameter for predicting emulsion type. For instance, while O/W emulsions are preferably formed with hydrophilic surfactants, W/O emulsions occur in the presence of hydrophobic emulsifiers [15]. Each emulsion type has its own morphology and characteristics, being converted into another type upon a phase inversion process.

The aqueous phase contains inorganic salts such as chlorides, sulfates, and carbonates of various metals, which may contribute to the process of corrosion, incrustation formation, and catalyst deactivation [11]. Hence, demulsification processes are necessary to separate the oil phase from the aqueous phase and its associated inorganic salts [12]. Water-oil emulsion phase separation is conducted in three stages: flocculation, sedimentation, and coalescence [16].

### 2.1. Formation and Stability of Emulsions

Visually, emulsions are a single-phase system produced by applying shear or sufficient energy to form dispersed droplets, regardless of the oil origin [17, 18]. However, being thermodynamically unstable, they tend to separate and return to the original two-phase condition. In this scenario, surfactants and stabilizers can make the emulsion kinetically stable for an extended period (occasionally for decades) [19]. Emulsion stability is impacted by the formation of an interfacial film around the droplets [8], which makes surface-active molecules line up at the interface, directing their hydrophilic portion to the aqueous phase and the hydrophobic portion to the oil phase. The interfacial layer formed by surfactants exhibits viscoelastic properties resistant to compression/deformation. Emulsion stability is also related to the mobility of the emulsifying species in the interfacial film [10].

Surfactants enable emulsification by reducing interfacial tension (IT) and forming a film at the water-oil interface, slowing the flocculation and coalescence of the droplets [20]. Resins and other polar compounds are examples of naturally occurring surfactants present in oil [20–22]. However, no consensus has been reached as to the role of asphaltenes in emulsion stability [23–25]. For example, asphaltenes and NAs form a rigid and well-structured interfacial film, wherein NAs salt effectively stabilizes the emulsion, while asphaltenes contribute only to the viscoelasticity of the interfacial film [26].

Mechanisms such as steric hindrance, electrostatic repulsion, and the Gibbs–Marangoni effect may likewise affect emulsions stability [27–30]. Steric hindrance forms a barrier that prevents coalescence between droplets, thus hindering their approximation. This barrier is generally constituted by surfactant molecules adsorbed at the droplet interface [27–30]. In turn, electrostatic repulsion occurs due to the existence of ionic surfactants at the water-oil interface, responsible for the formation of an electric double layer that causes repulsion and prevents droplet collision and coalescence [31]. This type of mechanism is insignificant in W/O emulsions due to the oil low dielectric constant, thus being way more common in O/W emulsions [8, 32]. The Gibbs–Marangoni effect, on the other hand, occurs due to the formation of interfacial tension gradients generated by adsorbed molecules, whose presence can lead to interfacial stress gradients capable of resisting tangential stresses. In practice, the droplets of an emulsion can be deformed and resist coalescence due to the elastic film formed [8]. For occurring in conjunction with other mechanisms, the Gibbs–Marangoni effect can be considered as a secondary mechanism for stabilizing emulsions.

The presence of solid particles can also provide kinetic stability to emulsions. Although not part of oil intrinsic composition, inorganic particles can act as barriers to prevent coalescence at the water-oil interface. The efficiency of these solids in stabilizing an emulsion depends on factors such as particle size (which should be much smaller than the drop size [33]) and wettability. These solids can also be electrically charged, improving the stability of the emulsion by electrostatic repulsion [8, 10, 34].

Inorganic solids commonly found in petroleum are derived from corrosion products (e.g., iron sulfide, oxides), mineral scale, formation sand, clay, drilling muds, and stimulation fluids. When incorporated into petroleum, these particles can aggregate with asphaltenes and resins, stabilizing emulsions [35, 36]. These inorganic compounds may also promote emulsion stabilization by interacting synergistically with NAs [37].

Oil composition is also important for emulsion formation and stability. Crude oil is a complex mixture composed mainly of hydrocarbons, corresponding to up to 90% (*wt*), and organic compounds containing small amounts of sulfur, nitrogen, oxygen, and organometallics [38]. Oil fractions can be classified as saturated, aromatic, resins, and asphaltene (components of the SARA fractionation), varying in solubility and adsorptive properties [39].

Asphaltenes and resins are the most polar fractions of crude oil, accounting for the most significant impact on emulsion stability [40]. These compounds exhibit acidic and basic functionalities responsible for stabilizing W/O emulsions [41]. Asphaltenes are insoluble in aliphatic solvents such as n-pentane and n-heptane but soluble in aromatic solvents such as toluene [42, 43]. These substances exhibit a polyaromatic core with aliphatic side chains containing heteroatoms such as nitrogen, oxygen, and sulfur and can coordinate with metals such as nickel, vanadium, and iron [42, 43]. Studies have shown that asphaltenes are strong stabilizers of W/O emulsions [44] and that their subfractions, called interfacially active asphaltenes, effectively contribute to emulsion stability [8, 24, 25, 45–50] by regulating the interfacial film viscoelastic properties [8, 26, 51, 52].

Asphaltenes have negligible surface activity and exhibit low emulsion stabilization when solubilized in oil [52]. These substances are estimated to first aggregate into colloidal particles, the actual interfacially active entities [53], forming asphaltenic aggregates (7–20 nm) that stabilize the interfacial film, thus promoting emulsion formation [54, 55]. Resins are responsible for maintaining colloidal stability by interacting with asphaltenic aggregates [56].

Resins comprehend the fraction insoluble in propane but soluble in n-pentane. They are classified as polar hydrocarbons with aliphatic characteristics but are less aromatic compared to asphaltenes. Despite having heteroatoms such as nitrogen (N), oxygen (O), and sulfur (S), they do not contain metals [42]. Due to their primary surface-active properties and secondary role in stabilizing the colloidal asphaltenic aggregates, resins are considered as an important class to forming stable emulsions, a fact supported by studies that found asphaltenes as a fine powder in oils with a low resin/asphaltene ratio [52, 57]. Other studies also suggest that resins delay asphaltenes migration onto the interface [8].

NAs are an important resin subgroup that is very effective in reducing IT [37]. Asphaltenes and NAs are present at the water-oil interface, synergistically stabilizing the emulsion by forming a viscoelastic film [37, 58, 59]. For example, the occurrence of hydrogen bonds makes the stabilization of asphaltenic aggregates with NAs likely to occur [58]. This is supported by the interfacial material characterization, which confirmed the predominance of carboxylic acids. For being also present in the asphaltene fraction, this finding suggests that asphaltene/acid films were responsible for stabilizing the emulsions [60, 61].

## 3. Naphthenic Acids (NAs)

NAs are naturally occurring compounds resulting from the biodegradation of petroleum, caused by bacteria or the lack of catagenesis (oil maturity level) in its deposit. Thus, NAs can be used as biomarkers and are directly related to oil maturity and biodegradation level [62, 63]. They can be present in different concentrations depending on the extraction site, reaching values ranging from 2 to 4% (*wt*) on average [64]. When investigating acidic oil from West Africa, most NAs were found in the light and intermediate fractions, while the latter fraction contained the NAs with the most surface activity [65]. NAs can also be found in PW in dissolved and dispersed forms, that is, as oil droplets [66].

**Figure 2:**
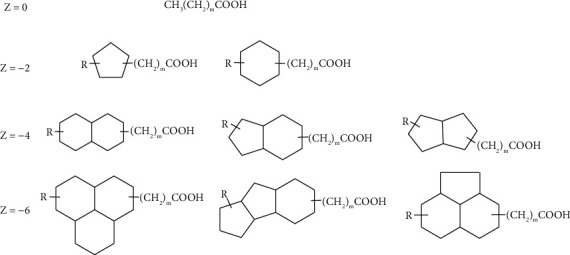
Typical naphthenic acid structure. The *Z* value refers to the hydrogen deficiency, *R* represents an alkyl substitute, and m represents the carbon chain size.

NAs consist of a mixture of linear- and branched-chains carboxylic acids and cyclic substituents [67]. The carboxyl group can occur directly bonded to the naphthenic structure or in the side chain, being separated by “ – CH_2_ – ” units [68].  Figure 2 shows the general structures of NAs, where m represents the number of “ – CH_2_ – ” units and *R* the aliphatic group [69].

The general chemical formula of an NA is C_*n*_H_2*n*_ + _*Z*_O_*x*_, where *n* indicates the number of carbons, *Z* refers to the hydrogen deficiency due to cyclic structures, and *x* is the number of oxygens in the molecule [5, 70, 71]. The *Z* value is related to the double bond equivalent (DBE) value, as shown by equations (1) and (2) [72], where C is the number of carbon atoms, H is the number of hydrogen atoms, and N is the number of nitrogen atoms of the molecule. Each element index in equation (1) is based on the element valence. Considering the general formula, the carbon index is [0.5 × (4 − 2)] = +1, the hydrogen index is [0.5 × (1 − 2)] = -0.5, and the nitrogen index is [0.5 × (3 − 2)] = +0.5. For the oxygen, the resulting index will be zero: [0.5 × (2 − 2)] = 0.

(1) $$\begin{array}{c} \text{DBE}=1+\text{C}-\frac{\text{H}}{2}+\frac{\text{N}}{2}, \end{array}$$

(2) $$\begin{array}{c} Z=-2\times \text{DBE}+2. \end{array}$$

The *Z* value is traditionally used for the qualitative analysis of NAs, indicating the presence of cyclic moieties such as acyclic (*Z* = 0), monocyclic (*Z* = −2), bicyclic (*Z* = −4), tricyclic (*Z* = −6), and tetracyclic (*Z* = −8) acids [73]. Cyclopentyl- and cyclohexyl-based acids (*Z* = −2) are the predominant structures commonly detected in oils, corresponding to approximately 95% of the NA composition. Conversely, the acids most found in oil sands process-affected water (OSPW) have 2 (Z = −4) or 3 (Z = −6) condensed rings [74, 75]. In PW, monocyclic acids are the main structures with *n* values of 7 to 12 carbons [76], and in OSPW the *n* and *Z* values range from 7 to 30 and from −12 to 0, respectively [67, 75, 77]. NA fraction can consist of hundreds of homologs and their isomers, thus posing an analytical challenge for qualitative and quantitative studies.

Although physical properties of NAs depend heavily on their chemical structure, average trends are provided for illustrative purposes. NAs and their sodium salts are generally water-soluble [62], but heavier homologs can precipitate under certain conditions. Smaller NAs are soluble in the aqueous phase at pH 5, whereas heavier NAs are often soluble in oil phase; heavier NAs become soluble in aqueous phase at higher pH values [8, 9]. These compounds' average density is between 0.97 and 0.99 g/cm^3^ [76], and their dissociation constant (pKa) ranges from 5 to 6 depending on the acid structure (for comparison purposes, the acetic acid and propionic acid exhibit pKa values of 4.7 and 4.9, respectively). NAs viscosity depends on the oil grade, being typically set between 40 and 100 mPa·s [78]. Moreover, these compounds exhibit low volatility, with Henry constants close to 8.56 × 10^−6^ atm·m^3^/mol and vapor pressure of approximately 2.35 × 10^−6^ atm, and their boiling point ranges between 250 and 350°C [79].

NAs are the class that most contributes to petroleum acidity [3]. The acid level is commonly measured in terms of total acid number (TAN) and expressed in milligrams of potassium hydroxide per gram of sample (mg KOH/g). Acidity can be measured by two different methods: the potentiometric (i.e., ASTM D664) and colorimetric protocols (i.e., ASTM D974) [80], with TAN values ≥0.5 mg KOH/g indicating high acidity [81]. The fact that oils with similar TAN values exhibited significantly different NA profiles is justified by the compositional complexity of NAs [82].

Besides being directly related to emulsion stability [65], NAs can also aid in foam formation in operational units and in the leaching of cations such as Ca^2+^ and Na^+^ during the desalination process, which can deactivate catalysts [83]. Despite their harmful effects on the oil refining process and on the environment, pure NAs and naphthenate salts are important raw materials in the chemical industry [69, 84].

## 4. Role of NAs in Stabilizing Emulsions

NAs play a major role in stabilizing emulsions [85], depending on the nature and distribution of the species, aqueous phase pH, and oil composition. Some studies have shown that smaller NAs are the most effective in regulating IT [86], while larger NAs stabilize emulsion by secondary mechanisms [12]. Systems formed by NAs/naphthenates/water produce the D-phase, composed of lyotropic liquid crystals. These aggregates are microstructures formed by several layers of surfactant and water, and their presence disproportionately increases emulsions stability [8, 31].

The ionic nature of the NAs significantly influences the emulsion interfacial properties [8]. When compared with neutral acids, the anionic state of acids has a more considerable impact on IT reduction [8], an effect related to the Donnan equilibrium, which occurs with an inversion of the solubility of the surface-active molecules from oil-soluble to water-soluble [87].

A study verified that W/O emulsion was predominant in the 6.5–14 pH range and 10.30–50% water contents (w/w) [65]. In turn, O/W emulsions predominantly occurred in a narrow high-stability region with 50% (w/w) water content and pH value between 12 and 13.5. NAs are found as naphthenates in pH values above 13, suggesting that O/W emulsions stability was secured by electrostatic repulsions between naphthenates present at the interface. Conversely, W/O emulsions stability is due to the asphaltenes and resins present in the heavy fraction of the oil [65].

The composition of NAs in pH-modulated interactions between NAs and asphaltenes was evaluated using a low TAN value oil [60]. Hydrophilic naphthenic and aromatic monoacids contributed to gel formation and promoted stable emulsions, whereas hydrophobic naphthenic and aromatic monoacids were insignificant to emulsion stabilization. Fatty acids adsorbed in paraffins formed a rigid interface. However, at neutral pH, these species competed with asphaltenes at the emulsion interface, thus decreasing the stability. Diprotic acids adsorbed at the interface and favored asphaltenes adsorption, producing very compact emulsions [60]. This finding was corroborated by yet another study, which found diprotic NAs and asphaltenes to form integrated films with high dilatational elasticity [61]. Following the same trend, a new method for isolating interfacial material (IM) revealed that interfacially active species comprised oxygenated acids and sulfur-containing compounds [45], and acidic compounds isolated from bitumen IM indicated the presence of O_3_S_1_, O_2_, and O_4_ species occurring as monoacids and diacids [88].

O/W emulsions were also found to be stabilized by a synergistic effect of the combination of the surfactant and the C_20_-C_30_ carboxylate salts in an alkalis/surfactant/polymer system with pH >8 [89]. In general, two types of naphthenates can be formed: calcium and sodium naphthenates. Whereas calcium naphthenates are present as solid deposits at the water/oil interface, sodium naphthenates promote emulsion stabilization [90]. The type of naphthenate formed will be determined by chemical nature of the NA [91], with calcium naphthenates being often associated with the presence of the tetraprotic naphthenic acid (ARN).

**Figure 3:**
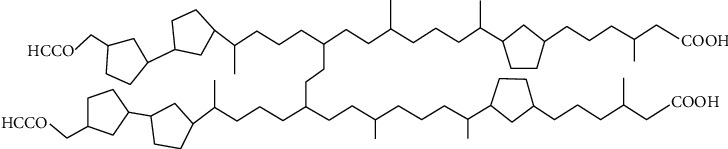
Chemical structure of tetraacid C_80_ (ARN).

ARN are a class of tetraprotic NAs that are typically found in low oil concentrations (0.6 to 3.6 ppm) [92, 93]. These acids include 4 to 8 rings in their structure, with the most abundant containing 6 rings with the molecular formula C_80_H_142_O_8_ (Figure 3). ARN is the predominant class of acids in calcium naphthenate deposits [92, 93]. The interaction of an ARN monolayer with calcium ions results in more robust and less soluble interfacial film, reducing its compressibility and promoting emulsion stability. ARN also exhibits dynamic self-association, producing elastic and solid films [94]. When compared with crystalline sodium naphthenates, calcium naphthenates form amorphous solids [94].

ARN preferably forms O/W emulsions with water content ranging from 10 to 90% (v/v) and pH interval from 4 to 10 [95]. The maximum emulsion stability was attained using intermediate water content and alkaline conditions. Moreover, NaCl and CaCl_2_ influenced the type of emulsion, whereby NaCl addition caused a phase inversion from O/W to W/O by increasing ARN affinity towards the oil phase. However, excessive concentrations of NaCl decreased emulsion stability. Ca^2+^ addition resulted in no phase inversion.

Besides the aforementioned conditions, the presence of other divalent cations (Mg^2+^, Ca^2+^, Sr^2+^, and Ba^2+^) decreased IT value due to the electrostatic attraction between cations in the aqueous phase and the naphthenates at the oil/water interface. This led to a higher density of NAs at the interface and the formation of positively charged monoacid complexes, which present high interfacial activity [96, 97].

Another study also evaluated the synergistic behavior of naphthenate-asphaltene mixtures [98]. The competitive adsorption at the oil/water interface between naphthenates and asphaltenes was described by two layers, in which naphthenates occupied the primary layer, reducing IT and controlling surface morphology, while asphaltenes were predominant in the secondary (or “floating”) layer.

## 5. NAs Techniques and Characterization

NAs consist of hundreds of different compounds, with more than one isomer for each *n* and *Z* value or compound class, making the separation and individual analysis of NA a difficult task [67]. A single mixture, for example, contains over 1,500 different NAs with a broad range of molar mass (up to 1,500 Da) [99–101]. Moreover, some species are detected at trace levels [88], requiring a preconcentration step prior to instrumental analysis. Numerous studies employed liquid-liquid (LLE) and solid-phase extraction (SPE) to extract NAs [88, 89, 102], but LLE was the most popular method. Sample preparation methods are vital for analyzing NAs by direct MS, enabling selective characterization and reducing ionic suppression effects [64, 103].

Over recent years, MS-based qualitative analysis became the most efficient analytical tool for the molecular analysis of NAs. As long as the mass spectrometer exhibits sufficient resolution and accuracy to provide reliable measurements of the monoisotopic mass and its corresponding isotopic patterns, any MS-based method can practically achieve group-type separation [64, 67, 75, 77, 99]. In this context, the direct-MS analysis offers an ideal platform for high-throughput experimentation, even though the analysis of individual compositional isomers and diastereomers may be jeopardized. Regarding diastereomers, hyphenated methods such as gas (GC-MS) and liquid chromatography (LC-MS) coupled to mass spectrometry are interesting alternatives for differentiating and quantifying isomers due to the improved chromatographic resolution and reduced matrix effect [102, 104–106].

### 5.1. Gas Chromatography-Based Methods

Gas chromatography (GC) is an instrumental technique widely used to separate mixtures based on the vapor pressure and solvation parameters of their composing molecules [107]. GC-MS is the most ubiquitous analytical technique for identifying and quantifying small organic substances in complex matrices [108]. Performed under high vacuum, electron ionization (EI) and chemical ionization (CI) are the standard ionization methods for GC-MS, being the most sensitive and robust analytical techniques. In theory, EI produces an odd-electron molecular ion ([M^+.^]) for detecting the nominal or monoisotopic mass [109]; however, the molecular ion stability depends heavily on the molecule chemical structure, so that not all molecules exhibit such an ion. Molecules with lower ionization energies tend to produce more stable molecular ions. Ionization energy increases in the following order: nonbonding electrons < *π* electrons < *σ* electrons. Hence, aromatic NAs will exhibit more intense peaks of the molecular ion compared to linear organic acids. Due to the excess of internal energy, molecular ion generation is also accompanied by fragmentation, thus making the EI spectrum energy-dependent. Furthermore, the EI mass spectrum is information-rich and reproducible, allowing for mass spectral databases.

An interesting approach to improve molecular ion detection is analyte derivatization. Pseudomolecular ions are available when using silylation reactions, yielding the even-electron [M-15]^+^ and [M-57]^+^ ions. Derivatization with *N*-methyl-*N*-(*t*-butyldimethylsilyl)trifluoroacetamide (MTBSTFA) generates *t*-butyldimethylsilylated (*t*-BDMS) acids, which produce stable [M-57]^+^ ions, where *M* represents the molecular mass of the derivatized acid [104]. Such pseudomolecular ions can be used for putative identification of the NAs, as shown in Table 1, where the [M-57]^+^ nominal mass is proposed based on the general formula C_*n*_H_2*n*_-_*Z*_O_2_ for alicyclic monocarboxylic acids [102]. Moreover, derivation methods such as methylation, acylation, and silylation [110–112] are used to increase analyte volatility and improve peak symmetry when using poly(dimethyl-diphenylsiloxane)-based GC stationary phases.

Although incipient in such applications, positive chemical ionization (PCI) is an interesting alternative for GC-MS analysis of NAs. PCI uses methane or ammonia as reagent gas, which provides soft ionization and generates the adduct ion ([M+H]^+^) depending on the analyte nature. For allowing an unequivocal detection of the adduct ion and its corresponding nominal (or monoisotopic) mass and isotopic pattern, PCI is an interesting method for assigning the elemental composition of complex mixtures.

GC-MS methods have been used to characterize NAs due to the hyphenated technique improved peak capacity [67, 75, 102, 113–117]. In a single run, a study managed to identify 156 NAs by GC-MS [67]. Similarly, when investigating the acidic fraction isolated from heavy gas oil of Marlim petroleum (Brazil) by GC-MS, another study detected alicyclic naphthenic acids with up to four rings (*Z* = −8) [102]. Authors also found NAs with carbon numbers ranging from 5 to 26 and *Z* values of 0 to −10 in marine sediments, with acyclic (*Z* = 0) and monocyclic carboxylic acids (*Z* = −2) with 12 to 19 carbons being the predominant NAs classes, corresponding to >70% of the total. Dicyclic- (*Z* = −4), tricyclic- (*Z* = −6), and tetracyclic (*Z* = −8) carboxylic acids were also reported by GC-MS analysis [115]. A different study found more than 100 NAs from OSPW, verifying extensive peak overlap with hydrocarbons [118], an unresolved complex mixture (UCM) also reported elsewhere [102, 117, 119].

To improve NA analysis, GC was coupled to Fourier transform ion cyclotron resonance (GC-FT-ICR MS) with different ionization methods, EI, CI, and atmospheric pressure chemical ionization (APCI) [120, 121]. Methane and ammonia were used as reagent gases [121], providing complementary information to previously reported methods. EI evidenced the pronounced UCM due to the occurrence of hydrocarbons, while CI improved the detection of N-containing species. Kendrick mass defect plots showed that O and O_2_ were among the most abundant classes, but HC, S, SO, SO_2_, O_3_, O_4_, N, NO, NO_2_, and NO_3_ were also present [120]. These results illustrate the potential of GC-MS analysis of NAs using high-resolution and accurate mass measurements to improve the molecular coverage of GC-based methods.

**Figure 4:**
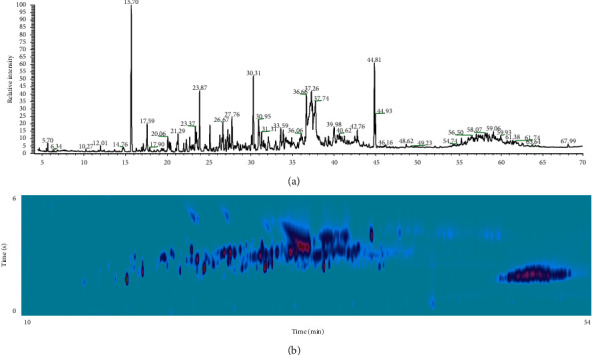
NAs chromatograms in simulated produced water sample obtained by GC-MS (a) and GC×GC-MS (b). GC analyses were performed on a TRACE1310 gas chromatograph coupled to a fast-scanning ISQ single transmission quadrupole mass spectrometer (Thermo Fisher Scientific, Waltham, MA, USA). For GC-MS, a 30 m × 0.25 mm-di (film thickness of 0.25 *μ*m) Equity1701 capillary column was used. The carrier gas was helium at a constant flow of 1.0 mL·min^−1^; 2.0 *μ*l of the sample was injected into a 250°C spitless injector. The oven temperature was set from 50°C to 260°C at 2 min^−1^. The ion source and transfer line were kept at 275°C and 260°C, respectively. Electron ionization (EI) was performed at 70 eV. Mass range was set from 35 to 350 Da at 6 scans s^−1^. For GC × GC-MS, the first-dimension column was a 20 m × 0.18 mm-di (0.18 µm film thickness) SLB-1MS capillary column and the second-dimension column was a 5 m × 0.25 mm-di (0.25 *μ*m film thickness) OV 1701 capillary column. The injector was operated in split mode with a 1 : 10 split ratio, at 250°C. Helium was used as carrier and auxiliary gas at constant flow rate of 0.5 and 20.0 mL·min^−1^. Modulation period was set to 6 s with a 400 ms reinjection (flush) pulse. The oven temperature was set from 50°C to 265°C at 5°C·min^−1^. Ion source and transfer line were kept at 275°C and 260°C, respectively. Mass range was set from 40 to 350 Da at 15 scans s^−1^.

In this context, comprehensive two-dimensional gas chromatography (GC × GC) is an interesting alternative for analyzing NAs in complex samples. This technique combines two sequential GC separation steps in a single analysis by using columns with complementary solvation properties [122–124]. Compared to conventional gas chromatography (1D-GC), GC × GC provides improved peak capacity [125], as shown in Figures 4(a) and 4(b). Moreover, such method performs group-type analysis of NAs more readily due to the structured chromatogram, wherein homologous series exhibit unique elution patterns.

GC × GC-MS has significantly improved the chromatographic resolution of NAs [126], facilitating the structural elucidation of individual isomers [6, 127–131]. The first GC × GC application dates from 2005, in commercial mixtures and bituminous sand samples, indicating acyclic and monocyclic acids containing one and two saturated rings [126]. The structured chromatogram allowed assigning identities according to the analyte *Z* value [132]. Two technical mixtures were evaluated (Sigma-Aldrich and Miracema-Nuodex), verifying the presence of NAs with *Z* = 0, −2, −4, and −6. More specifically, the carbon number of bicyclic acids (*Z* = −4) ranged from 9 to 16 [132]. Furthermore, an extensive series of tricyclic diamondoid acids and bicyclic, tricyclic, and pentacyclic diacids have been identified in OSPW [6, 127–131]. The distribution of diamondoid acid methyl esters was used to differentiate OSPW samples, also indicating their potential as petroleum markers [131]. NAs identification relied on the comparison of retention times and mass spectra with model compounds, but the use of retention indices is recommended to improve identification reliability.

Different classes of compounds have been identified in OSPW using GC × GC-MS. The identified carboxylic acids include indane, tetralin, cyclohexane, and adamantane [133]. Another work identified isomers of individual bicyclic aromatic acids in petroleum fractions using their methyl ester derivatives, suggesting that acids may be biotransformation products and indicative of bacterial processes occurring in reservoirs [134]. Other studies also confirmed the structures of some NAs of “nontraditional” chemical classes with reference standards, such as thiophene carboxylic acid (SO_2_ class) [133] and diamondoid dicarboxylic acids (O_4_ class) [127]. Identifying sulfur species is very useful for predicting and understanding the corrosion caused by NAs [133], while diacids formation in PW is probably due to late actions of the natural biodegradation on the corresponding monoacid [127, 133]. In another report, GC × GC-MS was used to monitor specific isomers of NAs in three geochemically distinct zones [135], successfully identifying a collection of acids like monocyclic, bicyclic, adamantane, and thiophene carboxylic (alkylated).

### 5.2. Liquid Chromatography-Based Methods

Liquid chromatography (LC) is a well-suited technique for analyzing polar analytes in complex samples, complementing the molecular coverage of GC-based methods. LC is considered extremely versatile by enabling multiple separation modes, such as normal phase (NP), reversed phase (RP), and hydrophilic interaction chromatography (HILIC) [136]. The most common ambient ionization methods in LC-MS are electrospray ionization (ESI), APCI, and atmospheric pressure photoionization (APPI). Among these, ESI is the most common ionization method used for NAs analysis by LC-MS, producing the even-electron [M+H]^+^ or [M-H]^−^ adduct ions [105, 137, 138]. Elemental analysis depends on the purity of the measured monoisotopic mass and its fine isotopic pattern. For instance, mass resolving powers of approximately 60,000 (at *m/z* 200) and 120,000 (at *m/z* 400) are required to resolve isobaric doublets with a mass difference of only 3.4 mDa [139].

Several studies in the literature have applied LC-MS, thus providing important information on NAs. This technique has been used, for example, for analyzing oxy-NAs (i.e., O_2_, O_3_, and O_4_) in OSPW samples [106], whereby O_2-_, O_3-_, and O_4_ classes accounted for 33.6% of total organic matter extracted. The results indicate that adding oxygen atoms in the molecules increased their pKa values, while double bonds and aromatic components reduced pKa values [106]. LC-MS was also applied to routine methods for quantitative analysis of classical NAs and oxy-NAs [105]. In this study, LC reduced ion suppression five-fold when compared with direct MS analysis, besides verifying significant variations in the response factors among the model NAs compounds [105]. Another approach employed LC coupled to sequential mass spectrometry (LC-MS/MS) [138, 140], thus allowing to resolve isomers based on retention time and the quantitation of individual NAs [140]. To improve (3-fold) and normalize NAs response factors, NAs were derivatized with *N*-(3-dimethylaminopropyl)-*N′*-ethylcarbodimide (EDC). The precursor ion scan was used to monitor the common product ion of the EDC derivatives, regardless of the original structure of the NA [138].

Analyzing NAs in OSPW samples by LC-MS shows that NAs concentration decreased after coke and ozone treatments. NAs structures were mainly composed of analytes with carbon number from 12 to 16 and *Z* values from −4 to −8, with only some NAs with carbon number above 18 [141]. Another study detected 55 unique NAs isomer groups in PW samples from different Norwegian offshore oil platforms with carbon number ranging from 8 to 26 and *Z* values of 0 to −18. Based on NAs distribution and the concentration in the samples, the C_8_H_14_O_2_ isomer group appeared to be an indicator of the presence of NAs in the samples [142].

A study conducted with wastewater samples collected from different wastewater treatment plants of petroleum refineries found NAs with *Z* values from 0 to −14 and carbon number from 10 to 20, with NAs with *Z* = −2 and −4 being the most abundant. Concentrations ranged from 113 to 392 *μ*g/L, with aromatic NAs estimated at 2 to 8%. Upon biological treatment, total NAs decreased by 65% and aromatic NAs by 86%. The removal mechanism for alicyclic and aromatic NAs was biodegradation via activated sludge, and polycyclic species NAs classes were recalcitrant to degradation [143].

LC-MS was used to characterize NAs in crude oils and refined petroleum products [144], verifying NAs with *Z* values ranging from −2 to −24 and carbon number from 6 to 60, among which those with *Z* = −2 to −6 were the most abundant. O_2_ was the most intense class among NAs, accounting for about half of the total acid component of the studied oils. In turn, O_4_ and O_6_ (presumably dicarboxylic and tricarboxylic acids) were observed only at very low abundances when compared with O_2_. Saturated carboxylic acids (*Z* = 0) were also detected in all studied samples in considerable concentrations [144]. In another study conducted with OSPW samples from marshy aquatic environments, NAs molecular characterization demonstrated O_2_, O_3_, O_4_, O_5_, O_6_, S, O_2_S, O_3_S, O_4_S, and O_5_S classes. Within this sample, O_*x*_ (*x* ≥ 2) was the predominant species, accounting for more than 90% of the total abundance, followed by O_x_S. Nitrogen-containing species were detected in very low abundance (≤0.4%) [145].

A different study used LC-MS to characterize samples of surface mining OSPW and *in situ* OSPW [146], finding the main classes of compounds to be O_1-6_, N, NO_1-4_, S, SO_1-4_, and NO_2_S. Class distributions were similar in both OSPW samples, but surface mining samples had higher prevalence of O_1-6_ species, while in situ samples showed relatively more N, NO_1-4_, S, SO_1-4_, and NO_2_S species. MS/MS experiments revealed that the O_2_ class included nonacidic compounds with dihydroxy, diketo, or ketohydroxy moieties [146]. In a subsequent study, the distinction between O_2_ species was performed in ozonated OSPW samples [147], obtaining a molecular profile information for O_*x*_ and SO_*x*_ species that indicated that species detected in ESI (+) mode were more recalcitrant to ozonation than those detected in ESI (−) ionization mode. The study highlighted the importance of using more than one ionization mode to improve molecular coverage, particularly for characterizing NAs by LC-MS [147].

A polar fraction of OSPW was used to assess the composition of NAs with LC-MS [148], whereby SO_3_ and SO_5_ species were the major classes detected by APCI (+). O_2_ class compounds with carbon number from 16 to 18 and DBE equal to 6 were the most prevalent, along with O_4_ classes, which are mainly bicyclic and tricyclic nonaromatic species, probably comprising mainly unesterified diacids with carbon number from 18 to 24 [148].

### 5.3. Direct Mass Spectrometry-Based Method

Direct MS analysis is possible by high-resolution mass analyzers such as FT-ICR MS and electrostatic ion trap with Fourier transform (FT-Orbitrap MS). Fourier transform-based MS instruments are key for petroleomics research, offering unprecedented mass resolving power and reliable compositional analysis [149]. More specifically, FT-ICR MS and FT-Orbitrap MS have outperformed contemporary time of flight-based MS instruments due to the achievable resolution and mass accuracy, even when a reduced number of ions is detected [150]. For example, an FT-Orbitrap MS was successfully used in NAs analysis, allowing for reliable group-type and semiquantitative analysis [151]. Another study endeavored to compare the performance of a compact high-field FT-Orbitrap MS to that of a gold-standard FT-ICR MS instrument, reporting similar compositional results in the FT-Orbitrap MS with average mass accuracies below 1 ppm [152]. Similarly, the FT-Orbitrap MS and FT-ICR MS were used for qualitative analysis of polar compounds in crude oil [153], finding the most abundant species in both instruments to be N, N_2_, O_3_, O_1_, O_2_, NO_2_, NS, and OS classes. While O_2_ class showed higher relative abundance in the FT-Orbitrap MS, the other classes were detected in lower proportions compared to FT-ICR MS [153].

The most common ionization methods for direct MS analysis include ESI [154], APCI [148], and APPI [155]. Such direct MS approach is also more prone to matrix effects, requiring careful sample preparation by online or offline fractionation depending on the complexity of the sample [156].

Before selecting the ionization method, one must carefully consider the desired classes of analytes to be measured. ESI is highly suitable for studying polar compounds containing heteroatoms, such as NAs [64]. Different studies have classified NAs from calcium and sodium naphthenate deposits by ESI (−)-FT-ICR MS [91, 154], verifying that sodium naphthenate deposits contained mainly linear saturated monoprotic NAs (C_15_ to C_35_), while calcium naphthenate deposits consisted mainly of ARN acids (C_80_), besides including also smaller species of ARN (C_60_-C_70_). Another study identified similar trends in crude oil samples from South America [93]. Moreover, APPI has been successfully used for the ionization of aliphatic and aromatic hydrocarbons [157], thus indicating that the combination of ESI and APPI can be used to provide complementary information about NAs classes in complex samples [155]. APPI allowed the ionization of additional compounds that were not detected by ESI, such as hydrocarbons, S_1_ and N_1_ [155]. When applied to the analysis of NAs from OSPW samples [71], APPI likewise enabled the identification of different classes that were not detected by ESI, such as hydrocarbons, N_1_O_1_, N_1_O_2_, N_1_O_3_, N_1_O_4_, N_1_O_5_, N_2_O_1_, N_2_O_2_, and N_2_O_3_. In negative mode (−), APPI also detected O_5_, O_6_, O_7_, and S_1_O_4_. Regarding O_2_ class, APPI (−) exhibited the same classes as ESI [71].

For enabling selective characterization and reducing the effects of ionic suppression, sample preparation methods are vital for the analysis of NAs by direct MS. In this context, FT-ICR MS was used to characterize NAs fractions in OSPW samples extracted with different solvents, namely, cyclohexane, dichloromethane, *n*-hexane, and cyclohexane: butyl acetate [103]. The solvent mixture cyclohexane: butyl acetate was the most effective, extracting a wide array of oxygen-containing species (O_*x*_, SO_*x*_, and NO_*x*_) and enabling the extraction of hydrocarbon species with five or more oxygen atoms. Compared to other solvents, the cyclohexane: butyl acetate mixture allowed the detection of NAs with a wider range of DBE values and carbon number and oxygen-containing classes up to O_9_, while n-hexane and cyclohexane extracted classes of compounds up to O_5_. Similar trends were observed for SO_*x*_ and NO_*x*_, suggesting that cyclohexane: butyl acetate was more efficient than n-hexane and cyclohexane for extracting polar compounds [103].

A study conducted with NAs present in a thermally degraded oil evaluated liquid-liquid extraction (LLE) and solid-phase extraction (SPE), finding SPE to allow the detection of a larger range of NAs species (*m/z* 200-1200), thus reducing the suppression of ions with higher molecular mass (*m/z* 700-1200 Da) [64]. LLE with hydroalcoholic alkaline solutions was also used to extract NAs that contributed to TAN variation. FT-ICR MS indicated species with less than 44 carbons and DBE values ranging from 3 to 4, suggesting chemical structures with a carboxylic acid and up to three naphthenic rings. Polar acidic species (O_2_ class), nitrogen-containing compounds, and NO_2_ compounds were also detected [158].

SPE and LLE were also evaluated under acidic and basic conditions to profile OSPW samples by FT-Orbitrap MS [159]. Oxygen-containing compounds (i.e., O_1-6_) were the most prevalent in acid extracts, especially O_4_. When extracted under basic conditions, classical NAs (O_2_) exhibited the highest intensity (ranging from 30 to 44%). A different study employed FT-Orbitrap MS to evaluate different solvents and SPE phases for the extraction of NAs in OSPW [160], verifying that NAs distribution depends on the extraction solvent/polarity. Hexane-based extraction was found to provide a more selective extraction of O_2_ species when compared with other solvents, such as chloroform and ethyl acetate. These findings indicate that, when comparing results from a sample, one must necessarily standardize the analytical method by employing the same extraction procedure.

Isolation of interfacial material requires dedicated sample preparation methods, such as fractionation using a modified aminopropyl silica (MAPS). FT-ICR MS analyses of interfacially active materials isolated by MAPS revealed a high abundance of low molecular weight molecules. All fractions presented O_3_S_1_, O_2_, and O_4_ species, as both monocharged and double-charged acids [88]. A study proposed a new isolation method for interfacial material, known as “wet silica,” revealing that isolated species contained an abundance of oxygen- and sulfur-containing compounds when compared with crude oil [45]. A compositional analysis by FT-ICR MS in an alkalis/surfactant/polymer system showed the presence of carboxylic acids, pyrroles, and phenols using MAPS and “wet silica” for the isolation of interfacial material [89].

Lastly, chemometric methods can be used to process compositional data, enabling the recognition of useful patterns and establishment of predictive models. For example, in a study conducted with partial least squares regression to estimate the TAN value of Brazilian crude oils [101, 161], most modelled compounds belonged to N and O_2_ classes, suggesting that O_2_ compounds were the main contributors to the observed TAN values. Such approaches are particularly interesting for highly complex mixtures, providing important information that may be disregarded in univariate analysis due to the high amount of data generated by high-resolution MS.

## 6. Concluding Remarks

This review showed that the stability of water-oil emulsions is related to the interfacial film formed between the continuous and the dispersed phases. To the best of our knowledge, the literature still lacks reports addressing oil-waters systems. Interface stability is improved by the occurrence of surface-active compounds naturally present in petroleum, where NAs are among the most important classes. NAs play an important role in the stability of oil emulsions by reducing the system interfacial tension according to the nature and distribution of NAs, among other factors. In this context, the development of reliable analytical methods for the qualitative and quantitative analysis of NAs is extremely necessary for improving our understanding of these compounds and their occurrence in crude oils. NAs composition is highly variable and depends on the oil source. To introduce the available solutions, significant developments using hyphenated MS methods and direct MS approaches were presented in this study, which also discussed basic aspects of sample preparation and data processing that accompany GC-MS, LC-MS, and MS.

We hope this review will raise awareness as to the existing challenges for improving our understanding on naphthenic acids. Despite the major advances in analytical methods for NAs, the elucidation of all NAs remains a critical research requirement. Such advances and their application to study naphthenic acids may provide a starting point for developing standard quantitative methods for characterizing NAs.

## Figures and Tables

**Table 1:** Expected even-electron ions ([M-57]^+^) for homologous series of *t*-butyldimethylsilylated naphthenic acids [104].

Carbon number (*n*)	Nominal mass for [M-57]^+^ ion						
	*Z* = 0	*Z* = −2	*Z* = −4	*Z* = −6	*Z* = −8	*Z* = −10	*Z* = −12
6	173	171	—	—	—	—	—
7	187	185	—	—	—	—	—
8	201	199	—	—	—	—	—
9	215	213	211	—	—	—	—
10	229	227	225	—	—	—	—
11	243	241	239	—	—	—	—
12	257	255	253	251	—	—	—
13	271	269	267	265	—	—	—
14	285	283	281	279	—	—	—
15	299	297	295	293	291	—	—
16	313	311	309	307	305	—	—
17	327	325	323	321	319	—	—
18	341	339	337	335	333	331	329
19	355	353	351	349	347	345	343
20	369	367	365	363	361	359	357
21	383	381	379	377	375	373	371
22	397	395	393	391	389	387	385
23	411	409	407	405	403	401	399
24	425	423	421	419	417	415	413
25	439	437	435	433	431	429	427
26	453	451	449	447	445	443	441
27	467	465	463	461	459	457	455
28	481	479	477	475	473	471	469
29	495	493	491	489	487	485	483
30	509	507	505	503	501	499	497
31	523	521	519	517	515	513	511
32	537	535	533	531	529	527	525
33	551	549	547	545	543	541	539

## Data Availability

No data were used to support the findings of this study.
